# Dear Sir

**Published:** 2011-02

**Authors:** Frank Otto Muller

**Affiliations:** Director: R&D, Integrow Health (Pty) Ltd, George, South Africa

## Dear Sir

The review article by Owira and Ojewole^1^ cites, among others, the influence of grapefruit (juice) on amiodarone pharmacokinetics and pharmacodynamics. Some apparent inconsistencies are worthy of note.

Amiodarone is not a ‘prodrug’ only. It has inherent pharmacodynamic effects. Its major N-dealkylation metabolite, N-desethylamiodarone (N-DEA) appears to possess even greater pharmacodynamic effects, notably with regard to cardiac electrophysiology.

The statement that concomitant ingestion of grapefruit juice ‘led to clinical prolongation of QT intervals and torsades de pointes’ is misleading and not substantiated by the articles cited.^2^,^3^ On the contrary, the grapefruit-initiated inhibition of N-DEA formation resulted in decreased cardiac electrophysiological effects. It is not clear whether ‘accumulation’ of amiodarone resulting from inhibition of conversion to N-DEA has clinical implications, but reduction in N-DEA concentrations may compromise the anti-arrhythmic action of amiodarone.

A further observation concerns the incorrect title of the article by Libersa *et al.*,^3^ which creates the impression that amiodarone metabolism is dramatically induced by grapefruit juice. The converse is true.

The authors of reference number 2 above (number 14 in the article) are incorrectly written. It should be Tsutomu U, instead of Urano T, and Ryuichi H, instead of Hasegawa R.
